# The Effect of Clinicopathological Features and Postoperative Management on Prognosis in Male Breast Cancer

**DOI:** 10.1155/tbj/2806453

**Published:** 2026-10-09

**Authors:** Shihe Liu, Qiying Song

**Affiliations:** ^1^ Department of General Surgery The First Medical Center of Chinese PLA General Hospital Beijing China 301hospital.com.cn

**Keywords:** hormone receptor, male breast cancer, postoperative chemotherapy, postoperative radiotherapy, prognosis, SEER database

## Abstract

**Background:**

Male breast cancer (MBC) represents a rare type of malignant tumor, comprising less than 1% of all breast cancer cases. Therapies for MBC patients are typically adapted from breast cancer treatments in women. This study systematically performed a retrospective evaluation of the clinicopathological features and prognostic determinants to offer evidence for a therapeutic strategy for MBC patients.

**Methods:**

A total of 29 MBC patients were selected from PLA General Hospital in China from 2006 to 2023 and 2589 patients were retrieved from the Surveillance, Epidemiology, and End Results database with their registration periods spanning from 2016 to 2020. The clinicopathological features between different groups were compared using Pearson’s chi‐square test (*χ* ^2^ test) or Fisher’ s exact test, as appropriate. Multivariate Cox proportional hazards regression analysis was utilized to assess the predictors of overall survival (OS). Subgroup analyses were further conducted to evaluate the therapeutic efficacy across different stages.

**Results:**

We identified 2618 patients totally. The primarily diagnosed median age of Chinese patients was younger than that of Western patients (59.8 years old vs 67.7 years old, *p* = 0.001). The infiltrating duct carcinoma was the most common pathological type for both groups. The subtypes (*p* = 0.622) and stages (*p* = 0.385) at diagnosis were similar between the two groups. Surgery at the primary site could improve the OS of MBC patients including Stage IV (*p* = 0.019). In stratified subgroup analyses, postoperative chemotherapy significantly improved OS in stages T2N1‐3M0 and T3‐4N1‐3M0 MBC patients. Postoperative radiation was associated with favorable OS in early‐stage (T1N0M0) and locally advanced (T3‐4N1M0 and T1‐4N2‐3M0) MBC patients.

**Conclusions:**

The median age at diagnosis for MBC patients in China was significantly younger compared to other populations. Postoperative chemotherapy significantly improved OS in patients with positive axillary lymph nodes and primary lesions exceeding 2 cm. Postoperative radiotherapy demonstrated survival benefits in partial early‐stage and locally advanced patients. Additionally, surgery at the primary site represented a viable therapeutic option for selected Stage IV patients.

**Trial Registration:** Chinese Clinical Trial: ChiCTR2500110646

## 1. Introduction

Male breast cancer (MBC), a rare malignancy, accounts for less than 1% of all breast cancer cases, and studies have documented a steady rise in its incidence over the past years [1]. MBC patients exhibit a less favorable prognosis compared to female breast cancer, with male patients demonstrating lower 5‐year survival rates and overall survival (OS) rates than those of female patients [2]. Therapeutic strategies for MBC patients are commonly extrapolated from treatments utilized for female breast cancer. However, MBC patients exhibit distinct clinicopathological characteristics compared to their female counterparts. This paper compared the clinicopathological features of MBC patients. Additionally, we performed a retrospective analysis of therapeutic outcomes to examine associations between different treatment modalities (surgical interventions, radiotherapy, and chemotherapy) and prognostic indicators across various stages. These findings provide evidence‐based guidance for optimizing clinical management of MBC patients.

## 2. Materials and Methods

### 2.1. Patient Population

The clinicopathological data of 29 male patients diagnosed with breast carcinoma were collected from January 1, 2006, to December 31, 2023, at the Chinese PLA General Hospital. The cohort exhibited an age distribution ranging from 28 to 84 years, with a median age of 61 years. This study additionally referenced data from the Surveillance, Epidemiology, and End Results (SEER) program, a population‐based oncology registry initiated by the United States National Cancer Institute and globally recognized as one of the most comprehensive publicly available cancer registries. Participants meeting any of the following exclusion criteria were excluded from the study cohort: 1) diagnosis of local disease at T0; 2) presence of other malignant tumors. The analytical cohort comprised clinicopathological data and prognostic information from 2589 male patients (≥ 18 years) who were diagnosed with breast cancer from 2016 to 2020. Among these, 2487 cases whose survival month was not 0 or unknown were included in survival analyses. All study participants provided written informed consent for inclusion in the SEER database prior to data collection.

### 2.2. Methods

We performed a comparative analysis of clinical characteristics between hospital‐identified MBC cases and the SEER database cohort. The clinicopathological variables encompassed age, laterality, tumor characteristics (TNM stage, histological type, estrogen receptor [ER] expression, progesterone receptor [PR] expression, and human epidermal growth factor receptor 2 [HER2] expression), therapeutic interventions (surgical interventions, radiotherapy, and chemotherapy), and prognostic outcomes (follow‐up duration and survival outcomes). Chemotherapy exclusively refers to cytotoxic chemotherapy regimens and does not include any form of endocrine therapy. The classification of chemotherapy was strictly based on the treatment codes and recorded systemic therapy categories in the SEER database and our hospital’s electronic medical records, which clearly distinguish cytotoxic chemotherapy from hormone therapy, targeted therapy, and other treatments. We performed a retrospective analysis using data extracted from the SEER database to evaluate stage‐stratified associations between therapeutic interventions and OS in MBC patients. Surgical intervention for breast cancer was defined as resection of the primary breast lesion. Histological classifications were divided into two subtypes: (1) infiltrating duct carcinoma and (2) other types which included lobular carcinoma, adenocarcinoma, papillary carcinoma, and other rare types of cancer. OS time (measured in months) was calculated from the confirmed diagnosis date to the endpoint. The primary endpoint was defined as the earlier occurrence of either death or the study’s predetermined cut‐off date.

### 2.3. Statistical Analyses

All statistical analyses were conducted using statistical software SPSS 25.0, GraphPad Prism 8, and Free Statistics software Version 1.9.2. Categorical data were presented as frequencies and percentages. Group comparisons were performed utilizing Pearson’s *χ* ^2^ test or Fisher’s exact test, as appropriate. OS was evaluated via Kaplan–Meier analysis, and independent prognostic factors were identified using multivariate Cox regression analysis. A subgroup analysis stratified by TNM stage was performed to evaluate the therapeutic effects of postoperative chemotherapy and radiotherapy. Hazard ratios (HRs) with corresponding 95% confidence intervals (CIs) were computed for all subgroups. Statistical significance was defined at *p* ≤ 0.05.

## 3. Results

### 3.1. Differences in Clinical and Pathological Characteristics

A total of 2618 patients were involved in this study, comprising 29 patients from the Chinese PLA General Hospital and 2589 patients from the SEER database (Table 1).

**TABLE 1:** Clinical characteristics of 2618 male breast cancer patients.

Clinical characteristics	Hospital in China (*n* = 29)	SEER (*n* = 2589)	*p* value
Age, mean ± SD	59.8 ± 12.9	67.7 ± 13.2	0.001
Range	28–84	18–90	
Average	59.4	67.8	
Middle age	61	68	
Laterality, *n* (%)			0.541
Left	16 (55.2)	1357 (52.5)	
Right	12 (41.4)	1175 (45.4)	
Bilateral	1 (3.4)	54 (2.1)	
Unknown	0	3	
T stage, *n* (%)			0.239
1‐2	24 (96)	2048 (86.4)	
3‐4	1 (4)	321 (13.6)	
Unknown	4	220	
N stage, *n* (%)			0.03
0	12 (48)	1357 (52.4)	
1	6 (24)	791 (30.6)	
2‐3	7 (28)	269 (10.4)	
Unknown	4	172	
M stage, *n* (%)			0.722
0	24 (96)	2303 (90.8)	
1	1 (4)	232 (9.2)	
Unknown	4	54	
TNM stage, *n* (%)			0.385
I‐II	17 (68)	1810 (75.5)	
III‐IV	8 (32)	587 (24.5)	
Unknown	4	192	
Histology, *n* (%)			0.01
Infiltrating duct carcinoma	23 (79.3)	2224 (93.5)	
Other	6 (20.7)	154 (6.5)	
Unknown	0	211	
Radiotherapy, *n* (%)			0.008
Yes	2 (6.9)	756 (29.2)	
No/unknown	27 (93.1)	1833 (70.8)	
Chemotherapy, *n* (%)			0.263
Yes	7 (24.1)	881 (34)	
No/unknown	22 (75.9)	1708 (66)	
Surgery, *n* (%)			0.293
Yes	26 (89.7)	2127 (82.2)	
No/unknown	3 (10.3)	462 (17.8)	
Subtype, *n* (%)			0.622
HR+/HER2−(Luminal A)	18 (81.8)	1996 (84.9)	
HR+/HER2+(Luminal B)	4 (18.2)	293 (12.5)	
HR−/HER2+(HER2 enriched)	0 (0)	12 (0.5)	
HR−/HER2−(TNBC)	0 (0)	49 (2.1)	
Unknown	7	239	
ER status, *n* (%)			0.125
Positive	20 (90.9)	2370 (97.3)	
Negative	2 (9.1)	67 (2.7)	
Unknown	7	152	
PR status, *n* (%)			> 0.999
Positive	20 (90.9)	2187 (90.2)	
Negative	2 (9.1)	237 (9.8)	
Unknown	7	165	
HER2 status, *n* (%)			0.518
Positive	4 (18.2)	305 (13)	
Negative	18 (81.8)	2047 (87)	
Unknown	7	237	

*Note:* HER‐2, human epidermal growth factor receptor 2.

Abbreviations: ER, estrogen receptor; HR, hormone receptor; PR, progesterone receptor; SD, standard deviation; TNBC, triple‐negative breast cancer.

Among the 29 patients in our hospital cohort, the mean age at initial diagnosis was 59.8 years (range: 28–84), approximately eight years younger than that of patients in the SEER database (mean age: 67.7 years; range: 18–90; *p* < 0.01). The predominant pathological type among patients in both groups was infiltrating duct carcinoma. However, the proportion of hospital patients diagnosed with other pathological subtypes (20.7%) was significantly higher than that recorded in the SEER database (6.5%; *p* = 0.01). The predominant molecular subtype among patients in both groups was Luminal A (HR+/HER2−), accounting for over 80% of cases. This was followed by Luminal type B (HR+/HER2+), which comprised approximately 10%–20% of cases. Over 90% of tumors exhibited ER positivity, with a similar proportion (approximately 90%) demonstrating PR positivity. In contrast, HER2 positivity was observed in only 10%–20% of cases. No statistically significant differences were observed between the two cohorts. At the time of initial diagnosis, patients categorized as Stages I‐II in the hospital constituted approximately 68% of the cohort, while those classified as Stages III‐IV accounted for about 32%. In contrast, the SEER database reported that approximately 75.5% of patients were classified as Stage I‐II, while 24.5% were categorized as Stage III‐IV. No statistically significant difference in TNM staging was observed between the two groups (*p* = 0.385); however, a significant discrepancy was noted in Grade N (*p* = 0.03). In total, 2 (6.9%) patients in the hospital cohort received radiation therapy, compared to 756 (29.2%) patients from the SEER database (*p* = 0.008). More than 80% of patients underwent surgical intervention (*p* = 0.293), and over 20% of patients received chemotherapy (*p* = 0.263). Among hospital patients, 26 (89.7%) patients underwent surgery, with modified radical mastectomy performed in 20 cases (69.0%) and total mastectomy in 3 cases (10.3%) (Table 2). The SEER database, however, lacked records of specific surgical methods for primary surgeries. Additionally, 21 (72.4%) patients presented with a painless breast mass in the breast, with a minority of cases accompanied by nipple discharge. The complete clinicopathological characteristics of the 29 patients from the hospital are detailed in Table 2.

**TABLE 2:** Other clinical characteristics of 29 male breast cancer patients from the hospital.

Clinical characteristics	Number (%)
Obesity	
Yes	11 (37.9)
No/unknown	18 (62.1)
Family history of tumors	
Yes	5 (17.2)
No/unknown	24 (82.8)
First symptom	
Breast mass	21 (72.4)
Other/unknown	8 (27.6)
Areolas	
Yes	14 (48.3)
No/unknown	15 (51.7)
SBR grade	
1‐2	12 (41.4)
3‐4	5 (17.2)
Unknown	12 (41.4)
Ki‐67	
< 15%	5 (17.2)
15%–30%	7 (24.1)
> 30%	9 (31)
Unknown	8 (27.6)
Breast surgery	
Standard radical mastectomy	2 (6.9)
Modified radical mastectomy	20 (69.0)
Simple mastectomy	3 (10.3)
Simple mastectomy + axillary sentinel lymph node biopsy	1 (3.5)
Other/unknown	3 (10.3)

Abbreviation: SBR, Scarff–Bloom–Richardson.

### 3.2. Survival Analysis for MBC Patients

This study aimed to identify independent prognostic factors in patients extracted from the SEER database. The median follow‐up duration was 26.2 months, with a range of 1–59 months. A total of 2487 patients with MBC were included in the multivariate Cox proportional hazards model (Table 3), with 420 mortality events. Finally, the multivariate Cox proportional hazards model incorporated the following variables: age, laterality, tumor stage, histological subtype, surgical interventions, radiotherapy, chemotherapy, and the statuses of ER, PR, and HER‐2. Multivariate analysis identified several independent predictors significantly associated with OS. Factors contributing to improved OS included the radiation therapy (HR 0.73; 95% CI, 0.56–0.95), chemotherapy (HR, 0.63; 95% CI, 0.49–0.82), and surgery (HR, 0.31; 95% CI, 0.24–0.4). Conversely, predictors of poorer OS comprised larger tumor sizes, specifically T2 (HR, 1.46; 95% CI, 1.14–1.88), T3 (HR, 2.47; 95% CI, 1.51–4.03), and T4 (HR, 2.28; 95% CI, 1.62–3.2). Additional adverse predictors included M1(HR, 1.82 95% CI, 1.35–2.46), high‐grade tumors with TNM stages II(HR, 1.33; 95% CI, 1–1.77), III(HR, 3.19; 95% CI, 2.27–4.49), IV(HR, 2.72; 95% CI, 1.87–3.96), subtype HR+/HER2+(HR, 1.46; 95% CI, 1.08–1.96), subtype HR−/HER2−(HR, 2.16; 95% CI, 1.21–3.88), HER‐2 positive (HR, 1.4; 95% CI, 1.05–1.87), and older age over 60 years (HR, 2.37; 95% CI, 1.82–3.1). Patients with ER positivity demonstrated a trend toward improved OS. Conversely, PR status was not identified as an independent prognostic factor for OS.

**TABLE 3:** Multivariate Cox regression model analysis of MBC patients.

Clinical characteristics	Number	Cox univariate analysis		Cox multivariate analysis	
		HR (95% CI)	*p* value	HR (95% CI)	*p* value
Age			< 0.001		< 0.001
≤ 60	646	1 (Ref)		1 (Ref)	
> 60	1841	2.38 (1.83∼3.09)	< 0.001	2.37 (1.82∼3.1)	< 0.001
Laterality			0.545		0.233
Right	1305	1 (Ref)		1 (Ref)	
Left	1147	0.86 (0.71∼1.04)	0.113	0.92 (0.76∼1.13)	0.43
Bilateral	32	3.65 (2.26∼5.89)	< 0.001	0.75 (0.42∼1.37)	0.354
Unknown	3	1.73 (0.24∼12.34)	0.584	0.53 (0.07∼4.02)	0.536
T stage			< 0.001		< 0.001
T1	990	1 (Ref)		1 (Ref)	
T2	1007	1.64 (1.28∼2.09)	< 0.001	1.46 (1.14∼1.88)	0.003
T3	89	2.9 (1.85∼4.55)	< 0.001	2.47 (1.51∼4.03)	< 0.001
T4	216	3.38 (2.51∼4.55)	< 0.001	2.28 (1.62∼3.2)	< 0.001
Unknown	185	4.63 (3.43∼6.26)	< 0.001	2.4 (1.54∼3.74)	< 0.001
N stage			< 0.001		0.897
N0	1316	1 (Ref)		1 (Ref)	
N1	773	1.24 (1∼1.55)	0.049	1.2 (0.95∼1.52)	0.121
N2	163	1.13 (0.77∼1.65)	0.532	0.97 (0.63∼1.48)	0.878
N3	97	1.51 (0.98∼2.33)	0.061	1.38 (0.86∼2.19)	0.178
Unknown	138	2.79 (2.05∼3.79)	< 0.001	0.99 (0.62∼1.59)	0.978
M stage			< 0.001		0.24
M0	2223	1 (Ref)		1 (Ref)	
M1	218	4.14 (3.31∼5.18)	< 0.001	1.82 (1.35∼2.46)	< 0.001
Unknown	46	2.4 (1.45∼3.96)	0.001	0.56 (0.3∼1.05)	0.071
TNM stage			< 0.001		0.244
I	692	1 (Ref)		1 (Ref)	
II	1074	1.18 (0.9∼1.56)	0.237	1.33 (1∼1.77)	0.05
III	344	1.91 (1.39∼2.62)	< 0.001	3.19 (2.27∼4.49)	< 0.001
IV	218	5.48 (4.07∼7.37)	< 0.001	2.72 (1.87∼3.96)	< 0.001
Unknown	159	3.36 (2.32∼4.84)	< 0.001	1.32 (0.85∼2.05)	0.224
Histology			0.043		0.622
Infiltrating ductular carcinoma	2157	1 (Ref)		1 (Ref)	
Adenocarcinoma	46	3.04 (1.94∼4.78)	< 0.001	0.74 (0.43∼1.28)	0.284
Ductal carcinoma	17	1.17 (0.37∼3.64)	0.79	1.69 (0.54∼5.3)	0.37
Papillary carcinoma	19	1.62 (0.67∼3.91)	0.287	1.38 (0.56∼3.4)	0.481
Carcinoma	34	4.04 (2.55∼6.42)	< 0.001	1.17 (0.7∼1.94)	0.545
Other	214	1.17 (0.84∼1.63)	0.353	1.04 (0.73∼1.5)	0.821
Radiotherapy			< 0.001		0.019
No/unknown	1746	1 (Ref)		1 (Ref)	
Yes	741	0.57 (0.45∼0.72)	< 0.001	0.73 (0.56∼0.95)	0.019
Chemotherapy			< 0.001		0.001
No/unknown	1621	1 (Ref)		1 (Ref)	
Yes	866	0.57 (0.46∼0.71)	< 0.001	0.63 (0.49∼0.82)	0.001
Surgery			< 0.001		< 0.001
No/unknown	408	1 (Ref)		1 (Ref)	
Yes	2079	0.16 (0.13∼0.19)	< 0.001	0.31 (0.24∼0.4)	< 0.001
Subtype			< 0.001		0.413
HR+/HER2−	1944	1 (Ref)		1 (Ref)	
HR+/HER2+	287	1.34 (1.01∼1.76)	0.041	1.46 (1.08∼1.96)	0.013
HR−/HER2+	12	1.64 (0.53∼5.12)	0.393	1.48 (0.46∼4.76)	0.506
HR−/HER2−	46	1.91 (1.09∼3.32)	0.023	2.16 (1.21∼3.88)	0.01
Unknown	198	2.19 (1.67∼2.86)	< 0.001	1.13 (0.83∼1.55)	0.428
ER status			< 0.001		0.908
Negative	63	1 (Ref)		1 (Ref)	
Positive	2309	0.51 (0.32∼0.8)	0.004	0.61 (0.35∼1.08)	0.089
Unknown	115	1.44 (0.84∼2.46)	0.188	0.78 (0.25∼2.44)	0.676
PR status			< 0.001		0.811
Negative	230	1 (Ref)		1 (Ref)	
Positive	2132	0.62 (0.47∼0.83)	0.001	0.78 (0.55∼1.11)	0.165
Unknown	125	1.83 (1.25∼2.68)	0.002	1.21 (0.43∼3.36)	0.717
HER2 status			< 0.001		0.178
Negative	1992	1 (Ref)		1 (Ref)	
Positive	299	1.31 (1∼1.72)	0.048	1.4 (1.05∼1.87)	0.02
Unknown	196	2.08 (1.58∼2.72)	< 0.001	0.69 (0.43∼1.11)	0.128

*Note:* HER‐2, human epidermal growth factor receptor 2.

Abbreviations: ER, estrogen receptor; HR, hormone receptor; PR, progesterone receptor.

### 3.3. Survival Analysis for MBC Patients With Different Treatments

Within the entire cohort, patients who received surgical intervention—either as a standalone treatment or combined with adjuvant radiotherapy or chemotherapy—exhibited significantly superior OS compared to nonsurgical patients. Additionally, a statistically significant difference in OS was found between patients who underwent surgery alone and those who received surgery combined with adjuvant radiotherapy or chemotherapy (*p* < 0.0001, Figure 1). We performed subgroup analyses stratified by tumor stage to investigate potential differences in surgical treatments (Supporting Table 1). Surgery demonstrated significant improvements in OS among patients at various stages: TNM I (HR, 0.19; 95% CI, 0.11–0.35), TNM II (HR, 0.22; 95% CI, 0.14–0.32), TNM III (HR, 0.18; 95% CI, 0.1–0.35), and TNM IV (HR, 0.51; 95% CI, 0.29–0.9). The univariate logistic regression analysis of patients from the SEER database revealed a significant negative correlation between ER status and radiotherapy (OR = 0.39; 95% CI = 0.21–0.73; *p* = 0.003). In contrast, no significant correlations were found between radiotherapy and PR (*p* = 0.531) and HER2 status (*p* = 0.164). Furthermore, a significant negative correlation was identified between chemotherapy and ER expression (OR = 0.34; 95% CI = 0.17–0.70; *p* = 0.003), while a significant positive correlation was observed between chemotherapy and HER2 expression (OR = 3.65; 95% CI = 2.71–4.92; *p* < 0.001) (Supporting Table 2). These findings reflected the influence of ER and HER2 status on treatment decisions.

**FIGURE 1:**
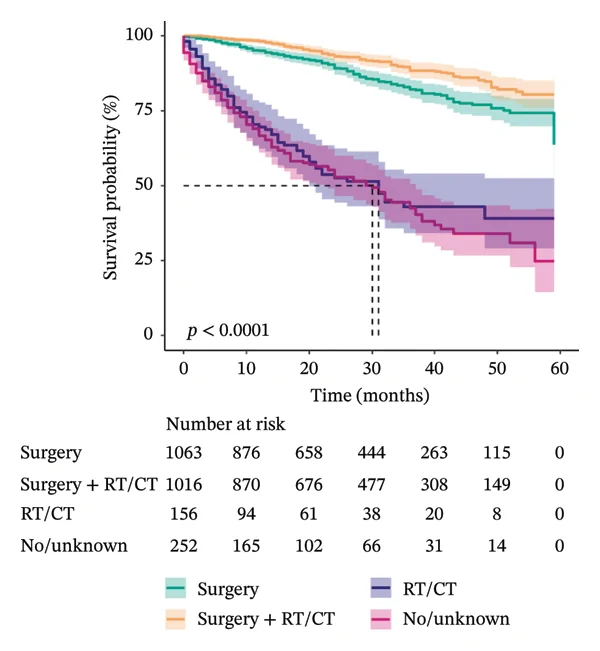
Kaplan–Meier curves for all patients with different treatments. RT = radiotherapy, CT = chemotherapy.

### 3.4. Survival Analysis Stratified by TNM Stages for MBC Patients With Radiotherapy and Chemotherapy After Surgery

In the surgery cohort, OS demonstrated significant differences between patients who received postoperative radiation therapy and those who did not (*p* < 0.01, Figure 2A). A similar disparity was observed for postoperative chemotherapy versus nonrecipients (*p* < 0.01, Figure 2B). The HR for death in the postoperative radiation therapy group and postoperative chemotherapy group compared to the control groups was 0.57 (95% CI: 0.4–0.8, *p* = 0.001) and 0.68 (95% CI: 0.47–0.97, *p* = 0.033) respectively, indicating a statistically significant improvement in OS.

**FIGURE 2:**
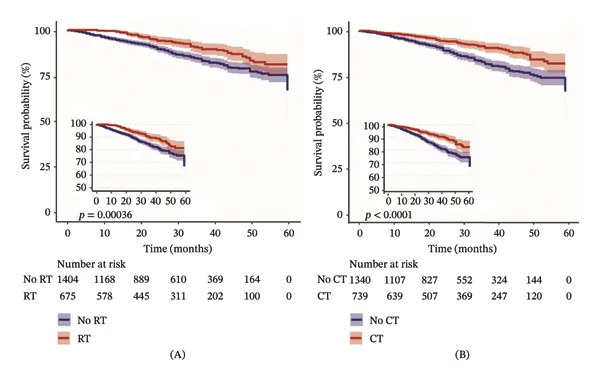
Kaplan–Meier curves for patients after surgery with or without postoperative radiotherapy or chemotherapy. (A) Postoperative radiotherapy and (B) postoperative chemotherapy. RT = radiotherapy, CT = chemotherapy.

To further investigate treatment response heterogeneity, we conducted subgroup analyses based on tumor stages, as detailed in Table 4. Postoperative radiation therapy demonstrated differential effects on OS across different TNM stages. Specifically, postoperative radiotherapy demonstrated a significant association with improved OS in patients at stages: T1N0M0 (HR, 0.11; 95% CI, 0.01–0.79), T3‐4N1M0 (HR, 0.17; 95% CI, 0.03–0.82), T1‐2N2‐3M0 (HR, 0.26; 95% CI, 0.1–0.67), and T2‐4N2‐3M0 (HR, 0.39; 95% CI, 0.17–0.85). Additionally, further analysis examining the association between postoperative chemotherapy and OS in patients at different stages (Table 5) indicated that postoperative chemotherapy was associated with favorable OS in patients at stages: T2N1M0 (HR, 0.22; 95% CI, 0.09–0.53), T2N1‐3M0 (HR, 0.38; 95% CI, 0.2–0.7), and T3‐4N1‐3M0 (HR, 0.36; 95% CI, 0.14–0.94), however, this benefit should be interpreted cautiously due to unmeasured confounding by endocrine therapy. Our findings indicated that postoperative chemotherapy was associated with favorable OS in patients with positive lymph node, and primary tumors exceeding 2 cm in size (HR, 0.36; 95% CI, 0.22–0.61), which may reflect combined effects.

**TABLE 4:** The correlation between postoperative radiotherapy and overall survival in male breast cancer patients with different TNM stages.

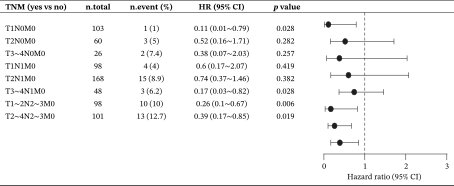

**TABLE 5:** The correlation between postoperative chemotherapy and overall survival in male breast cancer patients with different TNM stages.

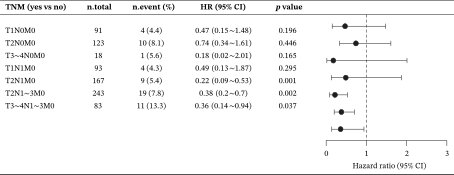

## 4. Discussion

This article presents a retrospective analysis comparing the clinicopathological data of 29 MBC patients from Chinese PLA General Hospital with that of 2625 MBC patients in the SEER database. The study further analyzed the impact of surgery, radiotherapy, and chemotherapy on OS in patients at different disease stages. The findings indicated that the average age of onset for MBC patients in China might be younger than that of their American counterparts. Surgical intervention not only enhanced OS for patients at Stages I–III but may also benefit those with Stage IV with metastasis for the primary lesion. Additionally, this study demonstrated that postmastectomy chemotherapy improved OS in patients with a primary lesion exceeding 5 cm and positive lymph nodes. Furthermore, postmastectomy radiotherapy was found to improve OS in MBC patients at early‐stage (T1N0M0) and locally advanced (T3‐4N1M0 and T1‐4N2‐3M0).

Recent reports indicate that MBC patients generally present at an older age than their female counterparts. [3, 4]. Among female breast cancer patients, the average age of Asian women, including those from China, is typically lower than that of Western women [5, 6]. A study analyzing 16,498 cases from the National Cancer Database (NCDB) found that the median age of the American MBC population was 63 years [7]. Our findings regarding age (average, 59.8; median, 61) are consistent with several small‐scale Chinese studies on MBC [8–10], which reported an average age of approximately 59 years, this average is younger compared to what has been observed in Western countries.

There are few reports on the correlation between the prognosis of MBC and clinical parameters. However, existing literature indicates that the statuses of HR and HER2 exert a substantial impact on the prognosis of MBC [11]. Approximately 90% of the MBC patients exhibit positive expression of ER and PR [3]. Specifically, the positivity rates of ER (91%–95%) and/or PR (80%–81%) in MBC [12] is notably higher than those reported in female breast cancer patients, where the positive rates for ER (77%–79.9%) and/or PR (64%–68.4%) are recorded [13, 14]. Furthermore, the expression of HER2 in MBC is generally regarded as lower than in females, where it is approximately 11% [14]. However, inconsistent findings in existing research indicate that the HER2 positive rate in female breast cancer is about 8.6% (91/1054) [15, 16], which is lower than the rate identified in our research. This discrepancy may be attributed to the accuracy of HER2 detection methods and the evolving detection standards. Most MBCs patients express sex hormone receptors, including ER‐positive, PR‐positive and HER‐2 positive. In contrast, triple‐negative breast cancer is relatively rare, occurring in approximately 0.3% of male cases [14].

Surgical interventions for MBC patients predominantly involve mastectomy, including simple mastectomy and modified radical mastectomy [17]. A retrospective study indicates that 81.6% of the 6919 MBC patients recorded in the SEER database from 1988 to 2017 underwent mastectomy [18]. Our study revealed that among 29 patients in the hospital, 89.7% (26/29) underwent mastectomy. Additionally, breast‐conserving surgery was identified as a prevalent surgical option. Findings from a real‐world meta‐analysis indicate that there is no statistically significant difference in OS between breast‐conserving surgery and total mastectomy (HR = 1.19, 95% CI 0.69–2.04) [19]. Nonetheless, total mastectomy continues to be the more widely recognized surgical method for MBC patients. The issue of whether primary tumor surgery is beneficial for Stage IV breast cancer patients has sparked considerable debate among both male and female patients [20, 21]. Our study suggested that primary lesion surgery could not only improve the OS of patients with early‐stage breast cancer but also offered benefits to OS in Stage IV MBC patients. A meta‐analysis investigating the association between primary tumor resection and prognosis in female patients with Stage IV breast cancer found that, primary tumor surgery might ameliorate OS, distant progression‐free survival, locoregional control, and the rate of no evidence of disease among patients in certain specific Stage IV [22]. However, considering that the surgery may have screened out Stage IV patients with better general conditions and who can tolerate the surgery, further prospective clinical trials are still needed.

Postmastectomy radiotherapy is typically administered to men with tumors larger than 5 cm or with four or more involved lymph nodes. Additionally, men with one to three involved lymph nodes, extensive lymphovascular invasion in the breast, or close surgical margins are also generally recommended to undergo postmastectomy radiotherapy [23]. The meta‐analysis results indicate that adjuvant radiotherapy after mastectomy significantly improved the OS (HR = 0.67, 95% CI 0.54–0.84), as well as the 5‐year OS, disease‐free survival, and local recurrence‐free survival of patients [19]. Abrams reported that adjuvant radiotherapy for MBC could enhance the 5‐year OS rates for patients with positive lymph nodes (in the group with 1–3 positive lymph nodes: 79% vs 72%, *p* = 0.05; in the group with 4 or more positive lymph nodes: 73% vs 53%, *p* < 0.001) [24]. Furthermore, findings from other studies indicated that adjuvant radiotherapy could improve local tumor progression and OS, particularly among patients with large tumors and positive lymph nodes [25, 26]. Our research results indicated that adjuvant radiotherapy after mastectomy in patients at T3‐4N1M0 (tumors larger than 5 cm) and T1‐4N2‐3M0 (with four or more involved lymph nodes) stages can improve OS. These findings align with previous studies that have demonstrated the beneficial effects of postoperative chemoradiotherapy on survival in various malignancies. Furthermore, our findings indicated that postoperative radiotherapy was a significant predictor of better OS in patients at stage T1N0M0. Currently, significant controversy exists regarding the role of postoperative radiotherapy in the management of early‐stage breast cancer. Compared with surgery alone, postoperative radiotherapy has been demonstrated to improve relapse‐free survival (RFS) in patients with early‐stage breast cancer [27], particularly as an integral part of breast‐conserving treatment [28]. From the perspective of health‐related quality of life (HRQOL), chemotherapy has little long‐term effect on patients with early‐stage breast cancer. The average HRQOL score experiences only a slight decline and returns to baseline levels or better within a few months [29]. However, recent studies indicate that adjuvant breast radiotherapy as a standard component of breast‐conserving therapy for early‐stage cancer may lead to overtreatment in many women. The PROSPECT study indicates that women with unifocal breast cancer diagnosed on MRI and with favorable pathological features may safely forgo radiotherapy [30]. However, the decision‐making for male patients is more complex due to the lack of specific data. Currently, postoperative adjuvant radiotherapy is not fully employed in MBC patients, with an average utilization rate of about 35% [31]. The lower rate of postoperative radiotherapy in the Chinese PLA General Hospital raises concern, and a potential explanation for this result is that some patients may have received follow‐up radiotherapy treatment at other facilities after surgery. Therefore, further investigations are essential to improve our understanding of radiotherapy in MBC.

Adjuvant chemotherapy is the standard treatment for many females with breast cancer. However, the role of adjuvant chemotherapy in MBC remains less defined due to the absence of large‐scale prospective clinical studies [32]. The clinical treatment is generally conducted with reference to the treatment guidelines for female breast cancer [33]. A study showed that the chemotherapy group exhibited a significantly higher 4‐year OS rate compared to the nonchemotherapy group (97.5% versus 95.2%, *p* < 0.001). In contrast, the 4‐year breast cancer‐specific survival rates were similar between the two groups (98% versus 98.8%, *p* = 0.128) [34]. However, several small‐sample studies present conflicting results regarding the potential benefits of chemotherapy for MBC. This inconsistency arises from multiple parameters that influence the decision to administer chemotherapy. A retrospective cohort study conducted in China involving 134 male patients revealed that adjuvant chemotherapy did not significantly improve the disease‐free survival time of patients, while it potentially improved the OS of patients with high recurrence risk factors [10]. In our study, we observed a higher proportion of patients with advanced clinical stages or HER2‐positive status in the chemotherapy group compared to the nonchemotherapy group. This disparity may explain why chemotherapy did not produce favorable outcomes in the previously mentioned research. Our results show that adjuvant chemotherapy was associated with favorable OS in patients with positive lymph nodes and primary tumor larger than 2 cm, consistent with established guidelines for adjuvant chemotherapy in female breast cancer.

This study is a retrospective observational study, and as such, inherent biases including information bias and confounding bias are unavoidable. The formulation of postoperative adjuvant therapy (chemotherapy/radiotherapy) regimens for MBC patients was guided by clinical practice rather than random allocation, a factor that may give rise to confounding by indication. Specifically, patients with favorable general status or low tumor burden were more likely to receive aggressive adjuvant therapeutic interventions. While multivariate Cox regression analysis was applied to adjust for major confounding factors such as age, TNM stage, and molecular subtype, unmeasured confounders including patient performance status, comorbidities, and treatment compliance may still exert an impact on the observed association between treatment modalities and OS. Moreover, we agree that the observed survival benefit associated with chemotherapy cannot be entirely isolated from the potential effect of concurrent or sequential endocrine therapy, since nearly 90% of patients in both cohorts were hormone receptor–positive and candidates for endocrine therapy. Therefore, the survival advantage in the chemotherapy group may partially reflect combined effects of cytotoxic chemotherapy plus endocrine therapy, rather than the benefit of chemotherapy alone. Additionally, the external validity and generalizability of the present findings are constrained by the single‐center setting of the Chinese hospital cohort, meaning the results may not fully reflect the clinical characteristics and treatment patterns of MBC patients in other regions of China, including southern China and rural areas.

## 5. Conclusion

In summary, our study indicates that the age of diagnosis for MBC patients in China may be younger than that of Western patients, while other clinicopathological characteristics exhibit similarities with those of foreign patients. Moreover, postoperative chemotherapy was associated with improved OS in patients with more positive node involvement and larger tumors. And postoperative radiotherapy can prolong OS in both early‐stage and locally advanced patients. Considering the distinct clinical characteristics of MBC patients in China compared to the SEER database, large‐scale, additional studies are necessary.

## Author Contributions

Shihe Liu and Qiying Song contributed to the study conception and design. Data collection, data analysis, and generation of tables and figures were performed by Shihe Liu. The first draft of the manuscript was written by Shihe Liu, and Qiying Song revised the manuscript critically.

## Funding

This research did not receive any specific grant from funding agencies in the public, commercial, or not‐for‐profit sectors.

## Disclosure

All authors read and approved the final manuscript.

## Ethics Statement

Approval to perform research on human subjects in this study was approved by the Ethics Committee of the First Medical Center of Chinese PLA General Hospital (S2024‐164). This was a retrospective study, and the requirement for informed consent was waived.

## Conflicts of Interest

The authors declare no conflicts of interest.

## Supporting Information

Additional supporting information can be found online in the Supporting Information section.

## Supporting information


**Supporting Information 1** Supporting Table 1 investigates the correlation between surgical interventions and survival rates in male breast cancer patients; Supporting Table 2 analyzes the relationship between hormone receptor expression and the effects of radiotherapy and chemotherapy, revealing a significant impact of ER and HER2.

**Supporting Information 2** STROBE Statement—Checklist of items that should be included in reports of cohort studies.

## Data Availability

The data that support the findings of this study are available from the corresponding author upon reasonable request. Publicly available datasets were analyzed in this study. These data are available at https://www.seer.cancer.gov/.
