# Supplementary material for: The Effect of Clinicopathological Features and Postoperative Management on Prognosis in Male Breast Cancer
Source: Breast J. 2026 Oct 9;2026:2806453. doi: 10.1155/tbj/2806453 (PMC13653141; doi:10.1155/tbj/2806453)
Supplement: Supplementary file 1 [file TBJ-2026-2806453-s002.zip › supplementary table1.docx]

**Supplementary table 1** The correlation between surgery and overall survival in male breast cancer patients with different TNM stages

Surgery demonstrated significant improvements in OS among patients at various stages: TNM Ⅰ (HR, 0.19; 95% CI, 0.11–0.35), TNM Ⅱ (HR, 0.22; 95% CI, 0.14–0.32), TNM Ⅲ (HR, 0.18; 95% CI, 0.1–0.35), and TNM Ⅳ (HR,0.51; 95% CI, 0.29–0.9).
