# Supplementary material for: The Effect of Clinicopathological Features and Postoperative Management on Prognosis in Male Breast Cancer
Source: Breast J. 2026 Oct 9;2026:2806453. doi: 10.1155/tbj/2806453 (PMC13653141; doi:10.1155/tbj/2806453)
Supplement: Supplementary file 1 [file TBJ-2026-2806453-s002.zip › supplementary table2.docx]

**Supplementary table 2** The correlation between the expression of hormone receptors and radiotherapy /chemotherapy

|  | Radiotherapy | | Chemotherapy | |
| --- | --- | --- | --- | --- |
|  | OR (95%CI) | *p* value | OR (95%CI) | *p* value |
| ER | 0.39 (0.21~0.73) | 0.003 | 0.34 (0.17~0.7) | 0.003 |
| PR | 1.12 (0.78~1.6) | 0.531 | 0.83 (0.57~1.2) | 0.319 |
| HER2 | 0.82 (0.62~1.09) | 0.164 | 3.65 (2.71~4.92) | <0.001 |

Abbreviations: ER, estrogen receptor; PR, progesterone receptor; HER-2, human epidermal growth factor receptor 2.
